# Variation of Cognitive Function During a Short Stay at Hypobaric Hypoxia Chamber (Altitude: 3842 M)

**DOI:** 10.3389/fphys.2019.00806

**Published:** 2019-06-26

**Authors:** D. De Bels, C. Pierrakos, A. Bruneteau, F. Reul, Q. Crevecoeur, N. Marrone, D. Vissenaeken, G. Borgers, C. Balestra, P. M. Honoré, S. Theunissen

**Affiliations:** ^1^Department of Intensive Care Medicine, Brugmann University Hospital, Brussels, Belgium; ^2^Unit of Oxygen Study, Translational Research Laboratory, Université Libre de Bruxelles, Brussels, Belgium; ^3^Laboratory of Integrative Physiology, Haute Ecole Bruxelles-Brabant, Brussels, Belgium; ^4^Faculty of Medicine, Université catholique de Louvain, Brussels, Belgium; ^5^Hypobaric Chamber, Queen Astrid Military Hospital, Brussels, Belgium

**Keywords:** acute hypoxia, cognitive function, PEBL, transcranial doppler, cerebral blood flow index

## Abstract

**Objective:**

To observe the effects of a fast-acute ascent to high altitude on brain cognitive function and transcranial doppler parameters in order to understand the physiological countermeasures of hypoxia.

**Methods:**

17 high-altitude-naïve male subjects (mean age was 26.3 ± 8.1 years) participated in the study. We measured Critical Flicker Fusion Frequency (CFFF), blood oxygen saturation, Psychology Experiment Building (PEBL) including three tests (Modified Math Processing Task, Perceptual Vigilance Task, and Time Estimation Task), as well as Cerebral Blood Flow index (CBFi), mean cerebral artery Systolic and diastolic velocities, Cerebral Pulsatility index (CPi), and heart Rate. All were measured at sea level, at least 1 h after arrival at the hypobaric hypoxia equivalent of 3842 m and 1 h after return to sea level.

**Results:**

Under acute exposure to hypobaric hypoxic conditions, significant decrease in CFFF [42.1 ± 1 vs. 43.5 ± 1.7 Hz at sea level (asl), *p* < 0.01], CBFi (611 ± 51 vs. 665 ± 71 asl, *p* < 0.01) and blood oxygen saturation (83 ± 4% vs. 98 ± 1% asl, *p* < 0.001) as compared to pre-ascent values were observed. Physiological countermeasures to hypoxia could be involved as there was no significant change in neuropsychometric tests, Systolic and Diastolic velocities and CPi. A significant increase in Heart Rate (81 ± 15 bpm vs. 66 ± 15 bpm asl, *p* < 0.001) was observed. All parameters returned to their basal values 1 h after regaining sea level.

**Conclusion:**

Hypoxia results in a decrease in CFFF, CBFi and oxygen saturation and in an increase in heart rate. As it decreased, Cerebral Blood Flow index does not seem to be the physiological measurement of choice to hypoxia explaining the maintenance of cognitive performance after acute exposure to hypobaric hypoxia and requires further investigation. Cerebral oxygen delivery and extraction could be one of the underlying mechanisms.

## Introduction

High altitude mountain environment causes important physiological stresses on the human body due to a decrease in atmospheric pressure with altitude inducing a low partial pressure of oxygen as well as low temperature (Imray et al., 2010).

Relationship between cerebral blood flow and partial arterial oxygen pressure (PaO_2_) is curvilinear whereas its relationship to arterial oxygen saturation (SpO_2_) is linear (Steinback and Poulin, 2016). Regional differences in cerebral artery blood flow exist. Anterior and mean cerebral arteries increase their flow with hypoxia whereas posterior arteries decrease their flows (Feddersen et al., 2015), which could have an incidence in cognitive functions (Lawley et al., 2017). Regulation of the cerebral blood flow is essentially linked to peripheral chemoreceptors (Iturriaga et al., 2016) and as the brain is highly sensitive to hypoxia (Lankford, 2014), physiological countermeasures are probably involved to compensate hypoxemia.

Literature shows mixed findings on cognition function under hyperoxic (Lafere et al., 2016; Brebeck et al., 2017; Germonpre et al., 2017) or hypobaric hypoxic conditions. This depends on duration of exposure, altitude level, individual susceptibility and/or education (Lefferts et al., 2019) and choice of battery testing. Decrease in reaction time (Li et al., 2000) or an increase in decisional errors (Davranche et al., 2016) has been reported. Furthermore prolonged exercise under hypoxic conditions is associated with a decrease in cerebral oxygenation partly associated with a decrease in cognitive functions (Dobashi et al., 2016). Working memory also seems altered under hypobaric hypoxic conditions (Malle et al., 2013). Cognitive performances are not only influenced by hypoxia but also through elements as age, gender and education (Lefferts et al., 2019).

Many studies have investigated cognitive performances after night acclimatization (Pun et al., 2018b) subacute or repeated exposure to high altitude (Pun et al., 2018a). Most showed a certain degree of cognitive impairment. Increasingly easy access to moderate or high altitude (cable car) has facilitated tourist to go there fast and without acclimatization. Certainty of potential danger in decision making has to our best knowledge never been explored in this setting.

Therefore, the objective of our study was to evaluate cognitive function during these first hours of acute exposure to high altitude. Previously, a pilot study from our group in the French ski resort of Chamonix showed surprising results in cognitive testing. Indeed, psychometric testing showed no statistical differences when done in the resort (mean SpO_2_: 98%) and at 3842 m (mean SpO_2_: 80%). This probably means that physiological countermeasures are present at least in the first hours of acute exposure to hypobaric hypoxia (high altitude). Here, we investigate transcranial Doppler variables and cognitive function after a rapid ascent (20 min) and a short stay (4 h) at 3842 m and 1 h after a return to sea level. This study aims to avoid climbing effort, to limit confounding factors and focusing on the “passive” physiological acute adaptations.

## Materials and Methods

### Population

All experimental procedures, conducted in accordance with the Declaration of Helsinki (World Medical Association, 2013), were approved by the Academic Ethical Committee of Brussels (Brussels Alliance for Research and Higher Education, B200-2014-045) and written informed signed consent was sought. Seventeen Caucasian, well educated (Lefferts et al., 2019) male subjects, naïve to high altitude were included in the study. Their mean age was 26.3 ± 8.1 years. Height and weight were, respectively, 179.6 ± 6.6 cm and 79.5 ± 8.6 kg. Subjects needed to be in good physical shape, non-smoking and not high-level athletes. Subjects suffering from arterial hypertension or another cardiovascular disease were excluded from the study, so were subjects on cardio-active drugs. No antioxidants (dark chocolate, red wine, green tea, …) were permitted 8 h preceding and during the study (Engan et al., 2012).

### Protocol

**FIGURE 1:**
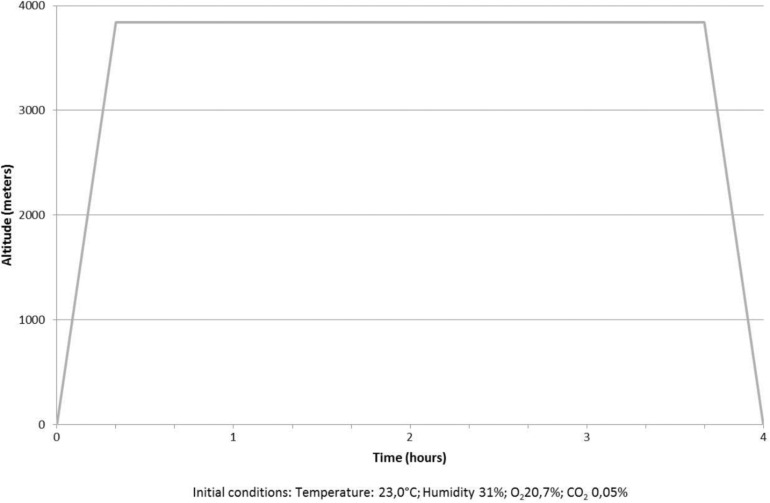
Ascending and Descending scheme.

Experiments were undertaken at two different dates (8 and 9 subjects) in the hypobaric chamber of Queen Astrid Military Hospital in Brussels, Belgium. The ascent scheme was the following to mimic the actual scheme present at Chamonix (France) to reach the top of the Aiguille du Midi pic: all subjects took the 20 min “cable car” ride from sea level to 3842 m; all two groups stayed 3 h 20 min at 3842 m before regaining sea level in 20 min. The purpose was to reproduce the scheme used in our pilot study in the French ski resort of Chamonix. All measurements were made before ascent, after at least 1 h stay at 3842 m and 1 h after return to sea level. Each experiment was made by the same investigator to decrease the risk of inter-experimenter variation. Measures were made in a thermoneutral space to exclude effects of cold. Exercise was restrained. Figure 1 shows the ascent scheme.

### Measures

#### Oxygen Saturation and Heart Rate

Blood oxygen saturation (SpO_2_) and heart rate, in beats per minute (bpm). were measured at the finger through an oximeter (Hand-Held Pulse Oximeter – CMS60F – Contec Medical Systems Co., LTD., Qinhuangdao, China) including an infrared light captor placed on the subject’s finger. It was expressed in %. The measure was fast (a few seconds).

#### Transcranial Doppler

Transcranial Doppler (TCD) was a noninvasive practical way to assess blood flow in cerebral arteries Blood velocity in the middle cerebral artery (VMCA) was measured with a 25-MHz TCD probe (Mindray M7, Mindray Bio-Medical Electronics, Shenzhen, China) going through the temporal bone window at both sides of the skull, at a depth of 4–6 cm for 10 s (Aaslid et al., 1982). The values of the brain side with the highest mean VMCA were registered. We calculated the pulsatility index [PI = (velocity systolic-velocity diastolic)/mean velocity] and cerebral blood flow index (CBFi = MAP × 10/1.47xPI), where MAP stands for mean arterial pressure (Pierrakos et al., 2013). The pulsatility index was a result of the interaction between the cerebral perfusion pressure, the amplitude of pulse arterial pressure, cerebrovascular resistances and heart rate (de Riva et al., 2012).

#### Cognitive Functions

##### Critical flicker fusion frequency

The CFFF was assessed with a specific device (Human Breathing Technology, Trieste, Italy). The device consists on a rotating ring, surrounding a short cylindrical waterproof plastic housing of 8 cm diameter containing the numeric (digital) frequency indicator covered by an acrylic transparent window. Attached to this housing a flexible cable is connected to a single blue LED (Light Emitting Diode, color temperature 8000 Kelvin), inserted in a small cylindrical waterproof container (to protect it from straying light and reflections). During the test the subject was looking straight at the LED light. The distance was adapted individually and freely to his personal best visual perception (generally around 50 cm). The investigator started at 35 Hz and increased or decreased the flickering frequency of the LED by steps of 0.25 Hz with the use of appropriate button. Thanks to the design of the device the subjects were not able to know the start and the finish frequency. When the subject saw a change in LED light from flicker to fusion, the subject acknowledged it to the investigator (through a sign without leaving sight of the LED) and the test was stopped. This noninvasive test has been validated to measure awareness (Hemelryck et al., 2013; Balestra et al., 2018; Rocco et al., 2019), mental fatigue (Ma et al., 2014), or executive dysfunction (Mewborn et al., 2015). It has already been demonstrated that CFFF could follow the recovery of cognitive function after propofol sedation earlier than psychometric testing (Sharma et al., 2011). This kind of correlation between mental state, CFFF and electroencephalography (EEG) has also been proposed recently in a world-class chess player, who showed parallel increases in CFFF threshold and theta Fz/alpha Pz ratio (Fuentes et al., 2018). In another recent study CFFF and attentional performance were closely related, with a tight relationship between the CFFF and occipital gamma band activity both in frequency and power (Kahlbrock et al., 2012).

This measurement has been presented in percentage as a relative variation of his personal value everyone being his own control.

##### Psychology experiment building language tests (PEBL)

Psychology experiment building language or PEBL is an open access software^1^ widely used in cognitive psychology (Li et al., 2000). It gathers dozens of functional tests. The PEBL tests were executed on a laptop. Three modified tests have been run, using the PEBL software package which was installed on the laptop. When the test chain is launched, detailed instructions are given. The results were automatically stored for not displaying any results to the subjects. Modified Math Processing Task, Perceptual Vigilance Task and Time Estimation Task were the three tests. These tests measured efficiency of higher brain functions such as processing speed, processing efficiency, working memory, concentration difficulties, and spatiotemporal integration.

In the Modified Math Processing Task (MMPT), the subjects are asked to solve several three-term additions and subtractions to determinate if the answer is greater or less than five. Two possible responses are proposed: “greater than” or “less than.” “Less than” responses are given by pressing the left shift key of the keyboard whereas “greater than” responses were made by pressing the right key of the keyboard. No result could be equal to five. The volunteer tried to complete the task as quickly and accurately as possible and responded correctly to every problem in a maximum of 4 s. The task ran for 3 min and stopped automatically. Correct, incorrect and timed-out results were stored for further analyses. This test permitted to evaluate participants in their capabilities associated to working memory, usually partially directed by the prefrontal cortex (Lee and Goto, 2015).

The Perceptual Vigilance Task (PVT) was a test used to measure a simple reaction time. This version was commonly used to measure sleepiness and arousal. The subjects had to press the spacebar, as quickly as possible when a red circle stimulus appears randomly in a 2 to 12 s delay. This happened 16 times. The RT (reaction time) was stored for further analyses. The test was highly sensitive to sleep loss (decrease in diurnal vigilance) (Thomann et al., 2014). Sustained attention seemed linked to frontal cortex (Loo et al., 2009).

In the Time Estimation Task (Time Wall), a target always started at the top of the screen and descended at a constant rate toward the bottom. 20 targets passed at different, but constant, speed. When the target exceeded two-thirds of the way down, it passed behind a wall and became invisible. Subject’s task was to press a key from the keyboard at the exact moment the moving target would pass through the notch marked at the very bottom of the display. In making this judgment, subject could not count or use any other rhythm method to facilitate his or her judgment. Subject followed the target with eyes and imagine it continuing straight down behind the wall to the notch. The real time compared to the estimated time by the subject was stored for further analyses. A precision score was established and calculated as followed mean estimated time minus mean real time divided by mean estimated time. Decision making and attention seemed related to the frontal cortex (Piper et al., 2015).

### Statistical Analysis

Statistical analysis was made with a Prism 6 statistical Software (Graphpad, La Jolla, CA, United States). Data are given as a percentage of pre-ascent values. The difference between the percentage of pre-ascent values and 100% (no change) was compared by a two-tailed one-sample *t*-test when normality of the sample was reached as assessed by the Kolmogorov-Smirnov test. Otherwise, the non-parametric Wilcoxon Rank Sum test was used. Statistically significant level was set at *p* < 0.05. Results are presented in the text as mean ± standard deviation.

## Results

### Oxygen Saturation and Heart Rate

Blood oxygen saturation significantly decreased at high altitude (83 ± 4%) as compared to pre-ascent (98 ± 1%, *p* < 0.001) and post-descent levels (98 ± 1%, *p* < 0.001). There was no difference between pre- and post-altitude values.

Heart rate significantly increased at high altitude (81 ± 15 bpm) as compared to pre-ascent (66 ± 15 bpm, *p* < 0.001) and post-descent levels (65 ± 12 bpm, *p* < 0.001). There was no difference between pre- and post-altitude values.

### Transcranial Doppler

**FIGURE 2:**
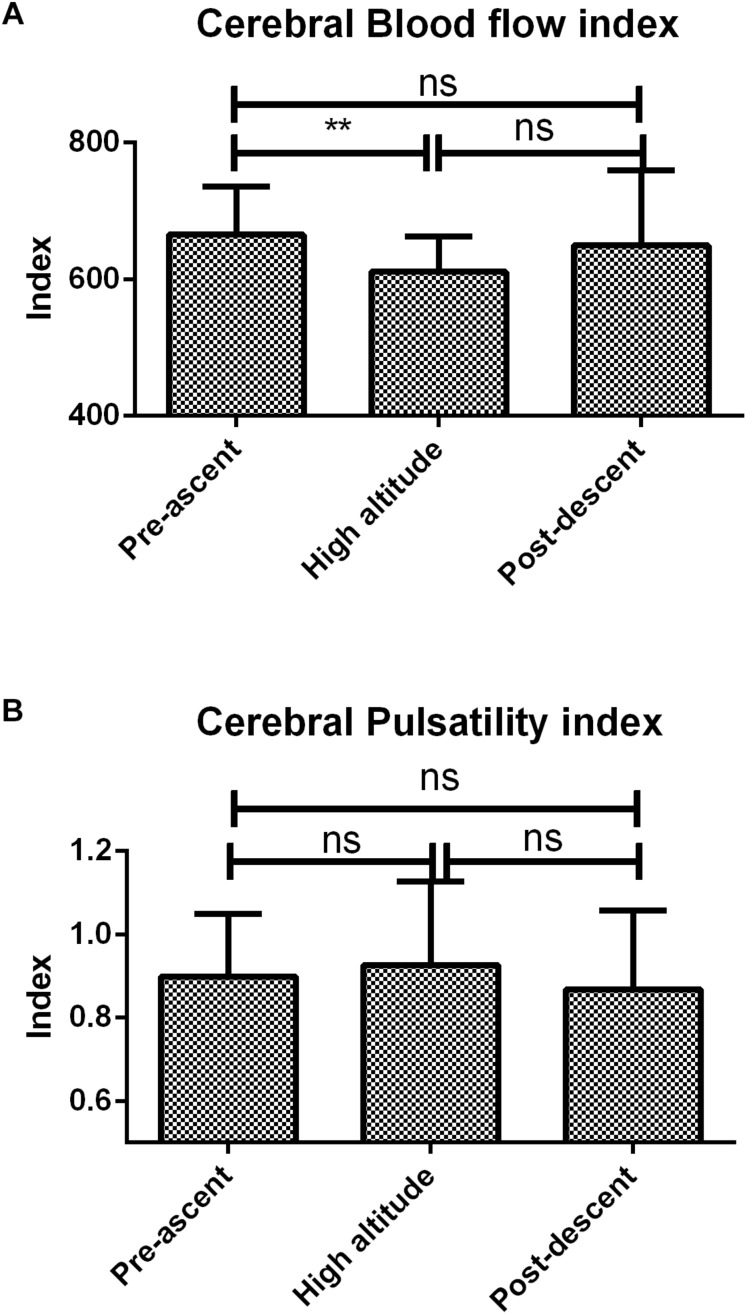
Mean cerebral artery velocity indexes. **(A)** Cerebral blood flow index, **(B)** Cerebral pulsatility index. Errors bars represent standard error of the mean (SEM). ^∗∗^*p* < 0.01.

**FIGURE 3:**
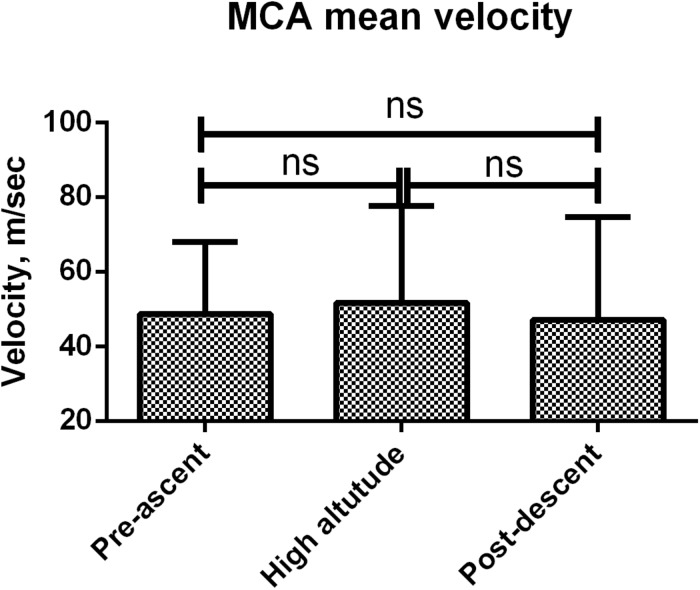
Mean cerebral artery mean velocity. Errors bars represent standard error of the mean (SEM).

Cerebral blood flow index decreased during high altitude stay (611 ± 51 vs. 665 ± 71 asl, *p* < 0.01) and returned to baseline 1 h after regaining sea level (Figure 2A). Cerebral pulsatility index did not vary throughout the experiment (Figure 2B). There were no statistical differences between systolic, diastolic or mean (Figure 3). velocities of the mean cerebral artery at different time frames.

### Cognitive Functions

#### Critical Flicker Fusion Frequency

**FIGURE 4:**
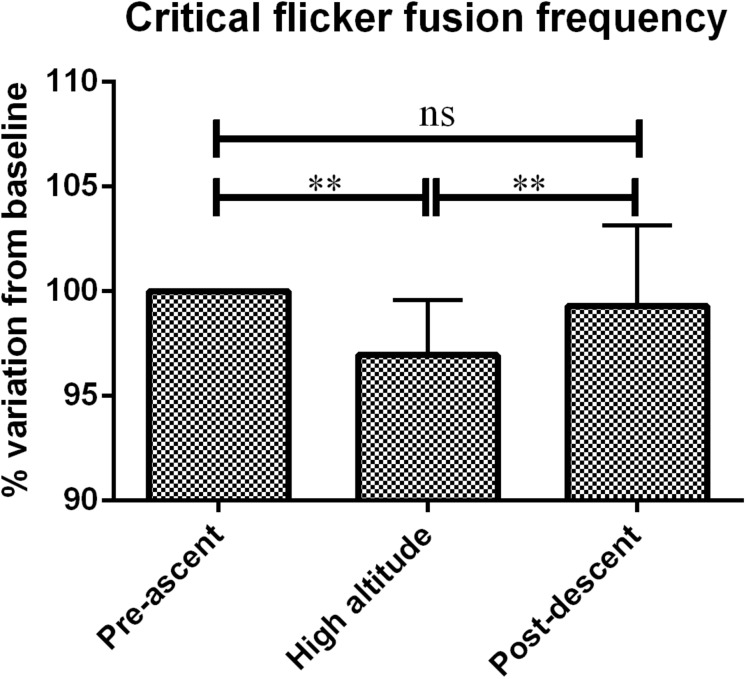
Critical flicker fusion frequency variation according to altitude expressed as percentage variation from baseline. Errors bars represent standard error of the mean (SEM). ^∗∗^*p* < 0.05.

Critical Flicker Fusion Frequency significantly decreased when measured at high altitude (42.1 ± 1 Hz) as compared to pre-ascent (43.5 ± 1.7 Hz, *p* < 0.01) and post-descent measures (43.1 ± 1.8 Hz, *p* < 0.01). Figure 4 shows CFFF values at different time frames.

#### PEBL

**FIGURE 5:**
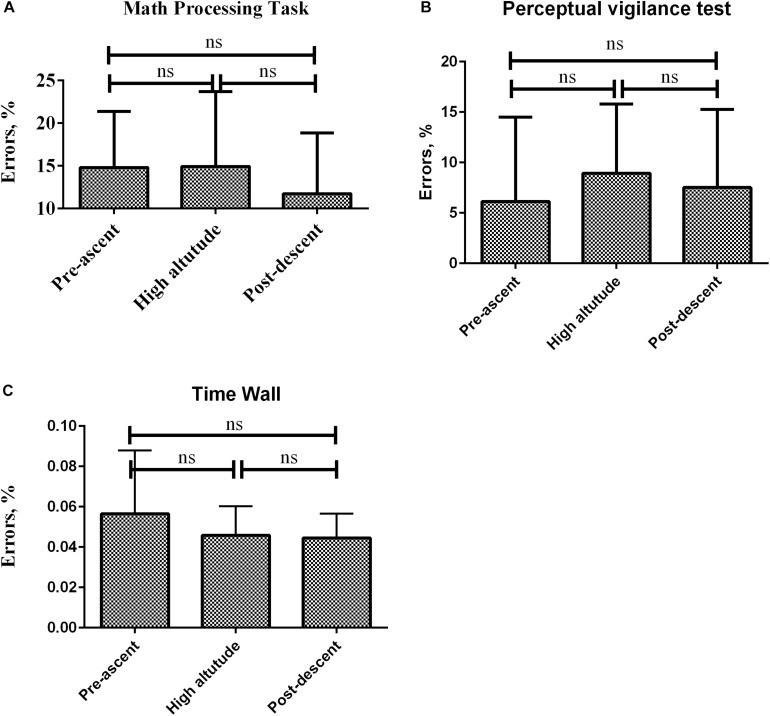
Psychology experiment building language tests variation according to altitude. **(A)** Math processing task. **(B)** Perceptual vigilance test. **(C)** Time wall. Errors bars represent standard error of the mean (SEM).

In the PEBL experiments, there was no statistical difference in all three tests between pre-ascent values, post-ascent values and high-altitude values as showed in Figure 5.

## Discussion

Under acute exposure to hypobaric hypoxic conditions, pulsed oxygen saturation decreased due to a decrease in atmospheric pressure with altitude. This was associated to an increase in heart rate, a physiological countermeasure. Our group showed a decrease in the critical flicker fusion frequency possibly associated to a decrease in arousal. This was linked with an unexpected small but significant decrease in the cerebral blood flow index excluding cerebral blood flow increase as a physiological countermeasure. There was no change in the performances of the Modified Math Processing Task, the Perceptual Vigilance Task and the Time Estimation Task, all part of the Psychology experiment building language tests probably meaning that no cognitive changes were seen in this time frame.

Pulsed oxygen saturation (SpO_2_) reflected the organism’s hypoxic state. Mean SpO_2_ significantly decreased at high altitude. This decrease was mainly due to a decrease in oxygen partial pressure in the breathing air (Imray et al., 2010). Acute ascent to high altitude prevented any metabolic acclimatization by the organism to compensate for decreased oxygen, temperature and pressure at high altitude, so SpO_2_ is a good indicator of the organism’s hypoxic state (Bilo et al., 2012).

The effects of effort/exercise, were not present avoiding some other compensatory mechanisms such as an increased Bohr effect or muscular metabolites build up, known to interfere with respiration (Balestra et al., 2011), nevertheless the complete absence of such mechanisms is not possible, we consider reducing them as much as possible.

Heart rate was increased, possibly by orthosympathetic stimulation through peripheral chemoreceptors (Iturriaga et al., 2016) triggering adrenergic neurotransmitters (Richalet, 2016).

No significant change was seen in systolic, diastolic and mean velocities of the mean cerebral artery (MCA) suggesting that there was no change in vascular diameter. If heart rate increases in high altitude, the absence of changes in MCA mean velocities would suggest an increase in blood flow. This was not what we observed; indeed, we saw a decrease in cerebral blood flow index.

Nevertheless, our results also show a significant decrease in critical flicker fusion frequency reflecting a decrease in cerebral arousal. This has also been seen in hypobaric chamber pilot testing (Truszczynski et al., 2009). Differences in CFFF without changes in psychometric tests could be explained by CFFF interrogating much lesser complex physiological pathways than psychometric tests (Hemelryck et al., 2013). Arousal is triggered by the stimulation of the ascending reticular substance (Jang et al., 2016) and it could be interrogated by CFFF. Furthermore, the decrease in CFFF could be due to decreased cerebral blood flow index Some similar results have been reported during acute orthostatic stimulations altering cerebral blood flow (Balestra et al., 2018). Similar results have been shown when using Cerebral Near Infrared Spectroscopy to assess brain’s oxygenation (Sakudo, 2016). This decrease in cerebral arousal even very early after acute exposure to high altitude should draw caution to unexperienced tourists (Debevec et al., 2019).

A decrease in SpO_2_ could induce cognitive dysfunction but there were no changes in psychometric testing. This means that cognitive function remains the same in acute hypobaric conditions, at least in the first few hours. Literature shows an increase in visual perception (Benedek et al., 2002), paving the way to hypoxic adaptation of the retina. Cognitive dysfunction has been incriminated as a symptom of acute mountain sickness (Issa et al., 2016).

Oxygen facilitates nerve conduction and interacts with GABA neurotransmission (Rostain and Lavoute, 2016). Recent data obtained from pilot trainees using magnetic resonance spectroscopy demonstrated that higher striatal concentrations of GABA and glutamate/glutamine were related to superior performance in action control allowing differentiation between high and normal performers (Yildiz et al., 2014). High arousal has been reported to decrease proactive control and increase reactive control compared to low arousal (Cudo et al., 2018).

Three hypotheses could explain the fact that we found no differences in PEBL during acute exposure to high altitude. First, the tests in the PEBL were maybe not exploring the more sensitive areas of the brain concerned with hypoxia such as areas involved in memory, accuracy and motor speed (Virues-Ortega et al., 2004) even though Time wall results did not differ at high altitude in our study. Second, complex reaction times could not be altered below higher altitudes (Virues-Ortega et al., 2004). This could explain why CFFF decreased without contemporary decrease of PEBL. Finally, no changes in PEBL could also mean that physiological countermeasures are engaged to maintain cognitive functions. Whether these are due to an increase in oxygen extraction or other mechanisms remains to be seen.

Most of the literature focuses on acclimatization, mainly from very high-altitude mountain expeditions. Recent literature examined cognitive function after acute, subacute and repeated exposure to high altitude (Pun et al., 2018a, b). These studies showed: (1) that acute exposure decreased psychomotor vigilance whereas a 6-day acclimatization prevented impairments during subsequent re-exposure (Pun et al., 2018b); (2) that attention was impaired at high-altitude but improved after acclimatization (Pun et al., 2018a). Sleep disturbance could be an important factor expanding these results (Sforza et al., 2004; Kim et al., 2007). Acute exposure in these articles were very different from our protocol. Indeed, Pun’s groups slept one night at moderate altitude (2950 m) and climbed to 5050 m each day for 1 week. In contrast with the existing literature where cognitive functions were altered (Roach et al., 2014; Davranche et al., 2016; Griva et al., 2017), our protocol focused on very rapid exposure to high altitude before any acclimatization is possible without any night sleep.

Limitations in our study include a limited number of participants, the absence of physical effort and the rather moderate altitude. Strengths include a rigorously controlled environmental conditions, homogeneity of our group, rapid ascent in altitude and the battery of cognitive tests used. The limited number of participants permitted the group to be homogeneous. Physical activity was not considered in the study as the purpose was to explore how tourists using cable cars manage their cognitive function at high altitude.

In our measurement setting, going from velocity to blood flow remains somehow speculative but CBF is nearly impossible to measure non-invasively. To assess cerebral vascular velocity, we used trancranial Doppler. This easy to use, noninvasive technique was transported into the hypobaric chamber. We used the cerebral blood flow index to approximate cerebral blood flow which is validated in septic critically ill patients (Pierrakos et al., 2013). In the Pierrakos trial, measures were made in hemodynamically stable patients. Compensatory hyperventilation inducing cerebral vasoconstriction could also explain a decrease in Cerebral blood flow index. Even if respiratory rate has not been measured in our study, literature is unanimous on this compensatory mechanism (Naeije, 2010; Paralikar and Paralikar, 2010). Some studies have shown an increase in cerebral blood flow in acute hypoxic conditions (Harris et al., 2013; Steinback and Poulin, 2016) even if contradictory data exists (Lawley et al., 2017).

This study is also interesting in a clinical intensive care setting where rapid profound hypoxia can occur very fast.

## Conclusion

Acute exposure to hypobaric hypoxia results in decreased oxygen saturation and increased heart rate. Critical flicker fusion frequency decreases at high altitude but Psychology experiment building language tests remain normal throughout the experiment. The decrease in the cerebral blood flow index does not explain the maintenance of cognitive performance and requires further investigation into other compensatory mechanisms such as increased oxygen extraction by brain tissue through near infrared spectroscopy. As all examined variables returned to normal 1 h after descent from high altitude, one could speculate that under acute conditions, critical flicker fusion frequency and cerebral blood flow index modifications may be transitory mechanisms.

## Data Availability

The raw data supporting the conclusions of this manuscript will be made available by the authors, without undue reservation, to any qualified researcher.

## Ethics Statement

All experimental procedures, conducted in accordance with the Declaration of Helsinki (World Medical Association, 2013), were approved by the Academic Ethical Committee of Brussels (Brussels Alliance for Research and Higher Education, B200-2013-127) and written informed signed consent, a total of seventeen healthy male subjects were included in the study.

## Author Contributions

DDB, CP, AB, FR, QC, NM, GB, DV, CB, PH, and ST made a substantial contributions to the conception or design of the work, acquisition, analysis, and interpretation of data for the work, drafted the work, and revised it critically for the important intellectual content and approved the final version of the manuscript and agreed to be accountable for all aspects of the work in ensuring that questions related to the accuracy or integrity of any part of the work are appropriately investigated and resolved.

## Conflict of Interest Statement

The authors declare that the research was conducted in the absence of any commercial or financial relationships that could be construed as a potential conflict of interest.

## Footnotes

1 http://pebl.sourceforge.net
